# Aging at Work: A Review of Recent Trends and Future Directions

**DOI:** 10.3390/ijerph17207659

**Published:** 2020-10-20

**Authors:** Jasmina Barakovic Husic, Francisco José Melero, Sabina Barakovic, Petre Lameski, Eftim Zdravevski, Petra Maresova, Ondrej Krejcar, Ivan Chorbev, Nuno M. Garcia, Vladimir Trajkovik

**Affiliations:** 1 Department of Telecommunications, Faculty of Electrical Engineering, University of Sarajevo, 71000 Sarajevo, Bosnia and Herzegovina; 2 Little Mama Labs, Gradacacka 29, 71000 Sarajevo, Bosnia and Herzegovina; barakovic.sabina@gmail.com; 3 Technological Centre of Furniture and Wood of the Region of Murcia (CETEM), C/Perales S/N, 30510 Yecla, Spain; fj.melero@cetem.es; 4 Telecommunication Networks Engineering Group, Technical University of Cartagena, 30202 Cartagena, Spain; 5 Faculty of Transport and Communications, University of Sarajevo, 71000 Sarajevo, Bosnia and Herzegovina; 6 Faculty of Computer Science and Engineering, Ss Cyril and Methodius University in Skopje, 1000 Skopje, North Macedonia; petre.lameski@finki.ukim.mk (P.L.); eftim.zdravevski@finki.ukim.mk (E.Z.); ivan.chorbev@finki.ukim.mk (I.C.); vladimir.trajkovik@finki.ukim.mk (V.T.); 7 Faculty of Informatics and Management, University of Hradec Kralove, 500 03 Hradec Kralove, Czech Republic; petra.maresova@uhk.cz (P.M.); ondrej.krejcar@uhk.cz (O.K.); 8 Instituto de Telecomunicações, Universidade da Beira Interior, 6201-001 Covilhã, Portugal; ngarcia@di.ubi.pt

**Keywords:** aging at work, discrimination, growth, deficit, assistance, policy, legislation

## Abstract

Demographic data suggest a rapid aging trend in the active workforce. The concept of aging at work comes from the urgent requirement to help the aging workforce of the contemporary industries to maintain productivity while achieving a work and private life balance. While there is plenty of research focusing on the aging population, current research activities on policies covering the concept of aging at work are limited and conceptually different. This paper aims to review publications on aging at work, which could lead to the creation of a framework that targets governmental decision-makers, the non-governmental sector, the private sector, and all of those who are responsible for the formulation of policies on aging at work. In August 2019 we searched for peer-reviewed articles in English that were indexed in PubMed, IEEE Xplore, and Springer and published between 2008 and 2019. The keywords included the following phrases: “successful aging at work”, “active aging at work”, “healthy aging at work”, “productive aging at work”, and “older adults at work”. A total of 47,330 publications were found through database searching, and 25,187 publications were screened. Afterwards, 7756 screened publications were excluded from the further analysis, and a total of 17,431 article abstracts were evaluated for inclusion. Finally, further qualitative analysis included 1375 articles, of which about 24 are discussed in this article. The most prominent works suggest policies that encourage life-long learning, and a workforce that comprises both younger and older workers, as well as gradual retirement.

## 1. Introduction

The older population is growing rapidly. In 2019, approximately 700 million people were aged 65 years or more in the world population. It is anticipated that this number will be doubled to 1.5 billion in 2050 [1]. During the next three decades, the size of the aging population in the European Union (EU) will have the same rising trend resulting in 149 million people in 2050 [2].

The growth of the older population is an effect of a reduced fertility rate and an increased life span [3]. Various factors influence the population aging, such as improved conditions in life and work, healthy lifestyles, and enhanced healthcare [4]. They lead to the growth of the aging population, which in turn, poses a broad range of economic challenges, including labour supply reduction and higher social costs. The aging population will continue to grow leading to a labor force reduction. This will lead to changes in the retirement age, an increased burden on government finances, and lower levels of pension provision [5].

Societies need to take a smart and multidimensional view of aging since these individuals may provide vast economic and social opportunities [6]. Rather than disrupting economic and social growth, the aging population may instead stimulate the social transformation of the 21st century, which can affect all sectors of society, including labour markets, the need for goods and services, as well as family structures and connections that affect several generations [2]. This process is focused on the transformation of the labour market by recognizing the aging population and its needs and capabilities. Older workers cannot perform certain jobs as a result of physical changes that progress with age [7]. Therefore, it is necessary to assess their current capabilities, modify their work positions, or employ them in a different position after appropriate training [8].

Not employing older people in certain work positions does not have to be a symptom of age-based discrimination, which is the most common form of discrimination, with one in five workers having witnessed it or personally experienced it [9]. It comes from a negative image of aging, preconceptions, and the overall perceptiveness of older workers themselves [10]. For example, there are stereotypes regarding the gender [11] or productivity of older people [12,13], the correlation between old age and illness [14], etc. Those stereotypes are sufficient motivation to look for a potential solution in policies and legislation that aim to inhibit discrimination, as well as promote an affirmative image of the aging labour force.

Sustainable growth at work means reaching the living and working conditions that support older people in their involvement in and continuance of work for a longer duration in their lifetime [15]. To achieve this, work has to be transformed to eliminate the factors that demotivate older workers to stay in or enter the labor market [16,17]. Concerns about the work sustainability, including the economic growth, pensions, and labour supply, have motivated a policy response as a potential solution to address the issues of longer working lives and later retirement [18,19].

Furthermore, an employment deficit calls for an effective response that should balance economic and regulatory needs with an influence on the jobs, working conditions, skills demands, and social protection [20]. Despite the many measures to cope with this issue [21,22], such as the unemployment benefits, social protection, and public employment services, activation of new policies is required as a potential solution to facilitate transitions to new jobs and decrease the risk of long-term unemployment and inactivity. Policies and legislation that encourage life-long learning seem to be an effective solution for preparing employees to change jobs if they cannot continue their current work due to decreasing opportunities [23].

In response to the aforementioned issues, the aging labour force has been studied over the last decade to contribute to the understanding of different motivations and solutions in the given context. However, there is a lack of systematic review of these motivations and solutions to support the aging labour force. Therefore, the aim of this paper is twofold. The first goal is to recognize and summarize the articles related to the aging labour force in terms of recent trends and future directions. The second objective is to capture the main motivation issues and solutions to support the concept of aging at work, i.e., the aging labour force.

The following research questions were posed:

1. What are the motivations that drive the research on the aging labour force?
2. What are the most common solutions for addressing the issues related to aging at work?

## 2. Methodology

### 2.1. Article Search Strategy

In order to answer the research questions, we examined studies on the aging labour force that were published between January 2008 and August 2019, to recognize the trends in the literature written in English with respect to motivation issues and potential solutions. We focused on the trends starting from the recession in 2008, when, although the economic growth slowed, the employment rate of older people remained strong, thus basically changing the position of older workers [24]. An additional motivation for focusing on this time period was because in the last decade, many assistive technologies have emerged that can aid older adults in different environments. At the same time, many jobs are transforming and can be successfully performed from home, which has recently become evident with the COVID-19 pandemic. Considering these two observations, the goal of this research is to investigate whether there is an underlying trend that reveals opportunities for aging at work.

We adopted the Preferred Reporting Items for Systematic Review and Meta-Analysis (PRISMA) methodology [25] to review the literature on aging at work policies. The PRISMA flow distinguishes separate stages of systematic reviews. These stages are the collection of papers, scanning of papers’ text, evaluation of eligibility of papers, and meta-analysis.

**Figure 1:**
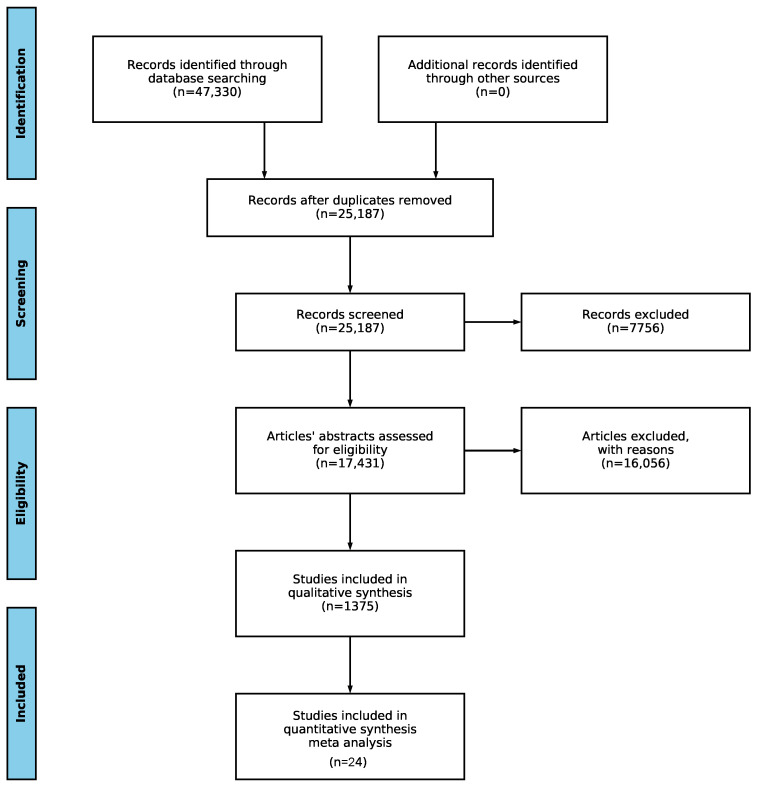
The PRISMA review workflow reflecting the number of articles identified, screened, processed and removed in each step.

The collected papers on aging at work policies exceeded the capacity that would allow articles to be searched manually. Thus, we used natural language processing (NLP) algorithms to perform an efficient search of the identified literature. The NLP toolkit [26] was designed to automate the literature search by using different search phrases, scanning, and evaluating eligibility within the PRISMA framework while generating visualizations of aggregate results. The NLP toolkit provides increased efficiency of the review process by screening the title and abstract while using the predetermined properties and their synonyms to determine the literature search phrases. It should be noted that the NLP toolkit does not understand the context and, therefore, categorizes more articles as relevant than a human reader would. However, it is a valuable resource that increases the efficiency of the review process, as demonstrated in a scoping review [27] that focused on wearable technology for connected health. The adopted PRISMA information flow is shown in Figure 1. Since the NLP toolkit automates the review process of publications that are indexed in only three digital libraries and because we have not taken into account the nonindexed publishers, some relevant publications (e.g., reference [28]) have been omitted from the analysis. This one and a few other papers were manually identified, and those publications originated from different digital libraries. They were used to confirm the findings of this review. However, we did not use these papers from other digital libraries to identify trends because the size of the searched digital libraries is sufficient for the purpose of the analysis.

**Figure 2:**
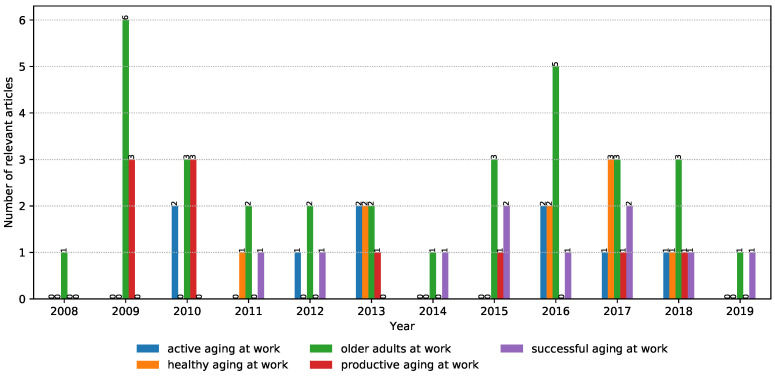
The number of identified relevant articles per year from January 2008 to August 2019.

**Figure 3:**
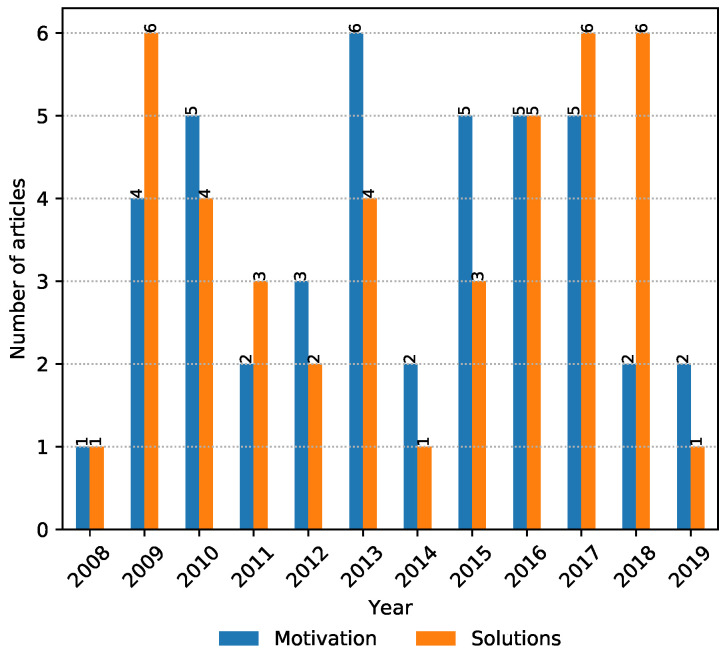
The number of relevant articles per property group and year within the period of interest.

**Figure 4:**
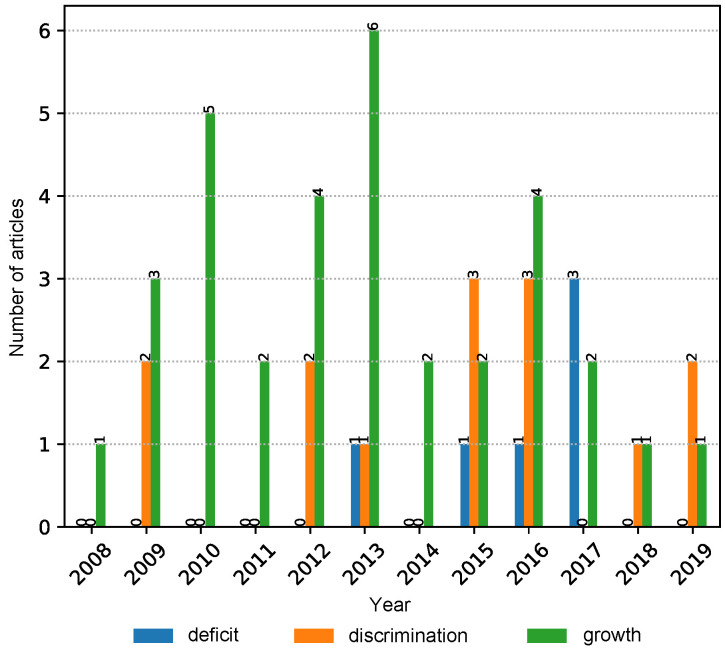
The number of relevant articles related to motivations property group categorized by “deficit”, “discrimination”, and “growth” properties from 2008 to 2019.

**Figure 5:**
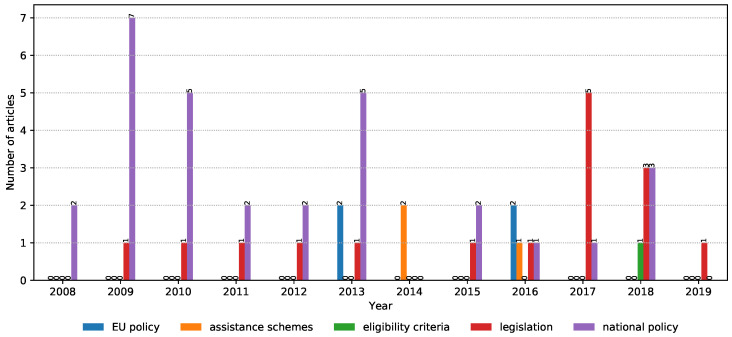
Solutions-related properties: “EU policy”, “assistance schemes”, “eligibility criteria”, “legislation”, and “national policy”. The trends apply to the period from 2008 to 2019.

The NLP search strategy was applied in order to automatically screen irrelevant articles that have a low correlation with the topics of interest in the study. Additionally, it helped in consolidating the collected articles by automatically merging results from multiple digital libraries as well as removing duplicate entries. Moreover, it allowed us to iteratively fine-tune and modify the search phrases in the hope of identifying more relevant articles. Finally, the NLP toolkit automatically generated charts (such as Figure 2, Figure 3, Figure 4 and Figure 5) that highlight the trends of publications for certain topics. For more details about the inner workings of the NLP-based toolkit, we refer interested readers to [26], and also to [27], which applied it to review wearable technology for connected health.

By using yearly graphs, we were able to analyze and report the potential trends in data by investigating articles in each property group (i.e., theme) separately.

The NLP toolkit input parameters are a collection of phrases. Keywords, together with their synonyms, are applied as search terms for the digital libraries used in the literature search. The input can be further expanded by NLP toolkit properties. Properties are phrases that are being searched within the title, abstract, or keywords section of the articles identified from the previous keywords search. Property groups are sets of properties that can be used for a more comprehensive presentation of search results.

The input parameters used in this study are shown in Table 1. These keywords, property groups and properties are the final versions after an iterative process in which all authors participated and considered different alternatives of keywords and properties, and analyzed the preliminary results. In the process of selecting articles to be included in the quantitative synthesis, four authors participated, of which at least two had to be in agreement.

### 2.2. Article Selection Process

The titles and abstracts retrieved by the NLP-based search strategy were evaluated by two independent researchers. They compared their opinions in order to select articles that satisfied the inclusion and exclusion criteria.

The inclusion criteria were as follows:

1. Articles that consider the concept of aging at work, i.e., the aging labour force.
  (a) Articles that discuss any of three motivation factors, i.e., discrimination, growth, and deficit;
  (b) Articles that support any of three solution pillars, i.e., assistance, policies, and legislation.
2. Articles that use research methodology with any results.

The exclusion criteria were as follows:

1. Articles that are about aging and older people in general that do not consider the concept of aging at work;
2. Articles that cover any of three motivation factors, i.e., discrimination, growth, and deficit, in a context other than the aging labour force;
3. Articles that cover any of three solution pillars, i.e., assistance, policies, and legislation, in the context other than the aging labour force;
4. Articles that do not provide sufficient information for classification.

When researchers differed in their opinions about an article’s suitability, the article was selected for further consideration. This resulted in an initial selection of 70 articles. Furthermore, the full texts of the chosen articles were reviewed in order to determine their suitability for further discussion. After the data abstraction of the final selected articles, two additional researchers separately reviewed 20% of randomly chosen articles. In the case of any disagreement on the suitability of articles, a third researcher was consulted for recommendation and assessment of the given article. This researcher was a specialist who drew a final conclusion regarding the article selection process.

For the selection of the final 24 articles, two of three authors needed to be in agreement, considering the completeness of the methods, relevance to the study goal, details about the population, and impact of the study.

### 2.3. Article Review and Analysis

We used the inductive approach for the article review and analysis. The selected articles were systematically organized into two groups:

1. Articles that focused on motivation factors (i.e., discrimination, growth, and deficit);
2. Articles that focused on solution pillars (i.e., assistance, policies, and legislation).

We generated a detailed summary of each article and extracted the following items: objective, methods, main findings, limitations, and keywords. The extracted items provided the input data for discussion and conclusions.

## 3. Results

After searching PubMed, IEEE Xplore, and Springer, we identified 47,330 potentially articles. After performing the PRISMA steps shown in Figure 1, the number of articles was reduced. Specifically, the removal of duplicates reduced the number to 25,187 studies. The first screening process eliminated an additional 7756 studies with an out-of-scope publication year, or other parsing issues (no title, abstract, etc.). Then, 17,431 papers were subject to the eligibility estimate using the automated NLP toolkit, which removed articles without any of the required properties. Eventually, 1375 papers remained as potentially relevant and eligible for further manual inspection. A total of 70 articles were initially selected to analyze the trends on the aging labour force, while 24 articles were used to explore the motivation issues and solutions in the given context.

### 3.1. Trends

The selected keywords aimed to show different aspects on the literature corpus on aging at work. Figure 2 presents the number of potentially relevant papers that contained the defined keywords and that were additionally filtered manually based on their relevance to the defined properties per year. A relatively similar number of identified articles can be observed in the evaluated time period. “Active aging at work” is the keyword with the smallest number of occurrences. The most frequent keyword phrase in the identified publications is “older adults at work”. The number of research articles did not grow in the period of interest, but articles that address the associated keywords seem to be distributed more evenly over time.

Findings related to property groups show that the number of papers related to “motivation” of the adult workforce is relatively constant, with a small decline in the last two years, while the papers focused on the “solutions” property group seems to be slightly more predominant in the last few years (Figure 3).

A more granular analysis was carried out on the property groups data at the properties level, and the chart reveals that “growth” is the primary topic within the motivation group of papers, followed by “discrimination”. The papers related to the topic of “deficit” appeared only in recent years (Figure 4).

The focus of papers within the “solutions” property group (Figure 5) seems to move from “national policy” based to “legislation”, while “assistant schemes” and “EU policies” seem to be of smaller interest for the scientific community. There was only one paper that addressed “eligibility criteria”, which makes this topic interesting for further research.

### 3.2. Motivations

A total of 12 articles out of 24 were selected for the further analysis of motivations that drive the research on the aging labour force. The selected articles were organized into three focus groups according to the considered terms related to motivation, i.e., “discrimination”, “growth”, and “deficit”. A more detailed analysis of these articles is presented in Table 2.

### 3.3. Solutions

The remaining 12 articles out of 24 were used for a more detailed analysis of solutions for the aging labour force. The selected articles were organized into three focus groups according to the considered solutions, i.e., assistance, policy, and legislation. Table 3 shows results of the analysis.

## 4. Discussion

### 4.1. Study Implications and Recommendations

The ageing labour force could represent a risk both for society and economy unless it is well managed. Therefore, the attention that researchers, governments and other stakeholders have devoted to this issue has grown over the time. According to analysis of motivations (Table 2) and solutions (Table 3) for ageing at work, possible policy implications have been identified and split into five parts:

Extend the length of work ability. Different organizations implement changes by creating common policies and strategies, but they are not oriented toward the older workforce. Intentionally interrupting the existing age-graded logic and its replacement with age-neutral logic are proposed in [16]. The authors in [29] found that the expected decline in employment could be partially offset by public policies that encourage the employment of older people. This causes problems for public finances due to expenditures on health, long-term care, pensions, etc. [3]. In order to encourage policies to maintain work ability at an old age, it is necessary to invest in decreasing of both work stress and social inequalities in health care [30]. However, extending the length of work ability does not just pose issues, but provides social and economic opportunities.

Avoid the age-based discrimination. The labour market will have to adapt working positions and eliminate the attitude of age-based discrimination, since it will have to fight for a working force older than 65 because it is lacking. When facing age-based discrimination at work, the organizational help and friends and family support were found to be significant in achieving better health and adaptability [31]. On the other hand, older workers with high job satisfaction without age-based discrimination remained longer in the labour market [32]. Finally, the authors in [10] found that experiences of discrimination were rare and reduced with age among men, whereas almost no age differences were noticed among women. This indicates that age-based discrimination is possibly overstated, and age-related obstacles could have been miscomprehended. Therefore, the flexibility of older workers can be seen as an opportunity for the active global aging trend [33].

**Table 2:** Detailed analysis of articles that focus on motivation factors.

Focus	Study	Objective	Methods	Main Findings	Limitations	Key Words
Discrimination	[32]	To recognize psychosocial work condition factors of interest to keep older workers by assessing the connection between the psychosocial work conditions and early voluntary pension.	Longitudinal study (survey). Study sample—general sample (N = 9913) aged 18-60 years, senior sample (N = 4477) aged 50 years, company sample (N = 3823) aged 18–80 years. Cox regression. Holm-Sidak correlation test.	Older workers with high job satisfaction, development possibilities, affirmative relations to management, and no age discrimination stayed longer in the work market. Positive relations with colleagues did not affect older workers decisions on early pension.	The measures were self-evaluated. The psychosocial factors were measured at single time point. Successive changes in the psychosocial work conditions could cause early pension that would be missed by the study.	Early pension, work conditions, management quality, job satisfaction
	[31]	To examine the relation between successful aging and stress sources at work among older workers in China	Questionnaire study. Study sample—242 workers aged >40 years. Method variance. Harman’s one-factor test. Factor analysis.	Perception of institutional support and social help from family and friends significantly corresponds to efficient aging at work.	Participants were surveyed at a single time point. The study relied on participants self-reports.	Successful aging, work stressor, social help, institutional support
	[10]	To improve comprehension of the discrimination at work, with a focus on age and gender challenges.	Survey study. Study sample—3203 workers with mean age 43 years. Computer-aided telephone interview. Binary logistic regression.	Daily discrimination was unusual. It appears with age among men, and not among women. The nature of work market age obstacles is not understood correctly, and the degree of aging discrimination is overstated.	There was a small number of workers who faced daily discrimination. The degree of daily discrimination has to be further investigated.	Ageism, employment discrimination, gender, work
	[33]	To investigate the age-related connection between job stress, extreme tiredness, prosperity, and associated personal, institutional, and community factors.	Survey study. Study sample—1298 participants aged 18 years or older. Descriptive statistics. Linear Regression. One-way analysis of variance.	Job stress was associated with several types of extreme tiredness and prosperity. Personal work style, institutional and community factors were associated with prosperity. Old age was connected to a poor perception of health.	The study did not compare work differences. The data were cross-sectional and the causal relation of the work conditions and style with job stress, extreme tiredness, and prosperity could not be confirmed.	Age difference, exhaustion, prosperity, work stress, work condition
Growth	[30]	To investigate the connection of social, demographic, economic and job related factors with disability.	Survey study. Study sample—2665 men, 2209 women aged 50–54 years. Principal component analysis. Confirmatory factor analysis. Poisson regression. Maximum likelihood estimation.	A decrease in job stress and sociable disproportion in healthcare is appropriate for the development of policies that support aging at work.	The disability indices were not formulated based on functional testing. The evaluation of stressful work was performed by abbreviated scales.	Socioeconomic position, aging workforce, work stress, work ability, social disproportion
	[16]	To examine organizational work disrupting age-graded policies.	Interview study. Study sample—23 organizations with employees aged 50–69 years. Qualitative content analysis.	Organizations implement changes by creating common policies and strategies, but not those oriented toward an aging workforce. They propose to intentionally interrupt the existing age-graded logic and replace it with age-neutral logic.	Creative, high-tech, or communications organizations were not studied. Sample size was small, so broader claims about Minnesota or U.S. workers cannot be made.	Organizational logic, older workers, pension, flexibility
	[29]	To examine the influence of demographic trends on the economic growth and employment level that Japan is expected to face in the next 20 years	NUPRI Macro Simulation model of the economy in Japan	The expected decline in employment could be partially offset by public policies that encourage the employment of older people.	Not reported.	Low fertility, population decline, population aging
	[3]	To provide a literature review on the need for the senior workforce and recognize main directions for research on this topic.	Systematic literature review. Empirical evidence.	There is a negative association between salary and employment outcomes for the senior workforce. The connection between efficiency and salary is defined by governmental conditions and motivation to take early pension.	The variations in micro-, macro-, and meso-level factors were not captured, simultaneously. There is a need for improvements in the analysis of the impact of age-based discrimination on the employing of older workers.	Work market, employment protection, regulation, legislation
Deficit	[17]	To examine the influence of organizational factors on work ability.	Cross-sectional study (online survey). Study sample—306 employees. Path analysis modeling. Maximum likelihood estimation.	Organizational culture and professional effort indirectly enabled the prediction of work ability, with job satisfaction mediating these relations.	The sample included mostly younger and female workers. The cross-sectional design of the study did not provide the possibility to understand causes and effects related to work ability.	Work ability, organizational culture
	[34]	To recognize professions prevailed by an older workforce and evaluate their vulnerability to hazards in these professions.	Survey study (interviews). Study sample—6502 workers aged 55 or more. Chi-squared test.	Work-related hazards should be decreased to inhibit professional disturbance in professions prevailed by an older workforce.	Self-informed data were included in the study.	Health issues, hazards, profession, musculoskeletal disorders
	[35]	To investigate job discrimination related to age and disability.	Equal Employment Opportunity Commission Integrated Mission System data from 1993 to 2007. Descriptive statistics.	Job discrimination of aged or disabled workers is focused on challenges involving seating, revenge, and cancellation.	Data do not contain supplemental information regarding a secondary cause for each filed allegation.	Job/age/disability discrimination
	[36]	To investigate the relation between psychosocial factors and pension intention of older employees, while considering healthiness and work ability.	Survey study. Study sample—3122 workers aged 50 years or older. Pearson correlation. Ordinal logistic regression.	Ageism and the absence of acknowledgement and growth opportunities are connected to older male workers’ pension intention. Work ability is strongly related to the pension intention of both genders.	The pension age could depend on unfamiliar alternations in the worker’s environment or health status.	Psychosocial factors, pension intention, healthiness, work ability

**Table 3:** Detailed analysis of articles that focus on solutions.

Focus	Study	Objective	Methods	Main Findings	Limitations	Key Words
Assistance	[37]	To critically review the literature on older farmers in Canada and the USA and describe how musculoskeletal disorders influence their ability to work.	Literature review. Twelve articles analyzed in detail.	Musculoskeletal disturbance can lead to trauma or loss of ability to farm. It is necessary to develop safer work practices and encourage healthiness, efficiency, and professional longevity.	Some related articles may have been excluded from the study due to the specificity of the search strings.	Older farmers, work-related musculoskeletal disorders, pension age
	[8]	To investigate the action plans that workers use to acquire skills in software and complete assignments	Exploratory study (interviews, surveys). Study sample—10 administrative assistants. Grounded Theory. Non-parametric statistics.	Administrative assistants are regularly communicating and sharing knowledge.	Exclusion of workers from different organizations, lack of extensive investigation on behavior at work, and creation of software tool design instructions.	Workplace, generations, collaboration
	[38]	To collect information to direct the preparation of programs for returning older adults to work	Survey study (questionnaires). Study sample—37 jobless participants aged 51–76 years. ANOVA. Chi-square test.	Participants who felt discriminated indicated the preference to acquire technological skills and get classroom-based education.	Work obstacles could not be generalized.	Older workers, absence of technological skills, work conditions, work experiences
Policy	[39]	To investigate factors related to perceived work ability in a sample of Brazilians sample aged 50 years and more	Longitudinal study (surveys). Study sample—8903 workers aged 50 years and over. Multivariate analysis. Poisson regression.	Work ability in old age depends on the life course, i.e., academic level, health conditions in younger and older age, minimum working age, etc. Policies aiming to extend longevity in the work market must consider these factors.	The collection of self-reported data associated with past experiences might have been affected by the preference to demonstrate an acceptable image, causing information bias. Establishment of temporal relations for the variable related to current conditions is limited.	Work ability, health, socioeconomic factors
	[40]	To review the documentation about the influence of psychological health on staying at work after pension and discuss consequences of public health policies.	Systematic literature review. Ten articles analyzed in detail.	Staying at work after pension can be positive for psychological health. Pension action plans are required to provide national policies that will increase the pension age and not exacerbate any disproportion in the older population.	Only cross-sectional and longitudinal studies investigating the impact of unexpected variables on psychological health were involved in the review.	Pension, job status, psychological health, social policy
	[7]	To analyze the literature on workplace health promotion (WHP) aimed at older workers	Systematic literature review. Eighteen articles analyzed in detail.	Existing documentation does not demonstrate that WHP enhance work ability, retention, efficiency, lifestyle, health, or prosperity of the senior workforce.	The heterogeneity and low quality of the studies makes it difficult to synthesize the literature and draw the conclusions.	Workplace health promotion, senior workforce, health, lifestyle
	[41]	To investigate the results of unfulfilled expectations of staying at work after age 62 on life satisfaction.	Longitudinal survey. Study sample—1684 workers aged 51 and over. Growth mixture modeling. Descriptive statistics. Linear regression. Multi-nominal logistic regression.	Majority of men and almost no women expected to stay at work after age 62. The subjective prosperity of older adults is affected by unmet expectations of staying longer at work.	The significance of different job options before full pension was not assessed.	Work expectations, pension, life satisfaction, subjective prosperity
	[42]	To find out whether the workers’ ages determine the evaluation of their work–life balance.	Survey study. Study sample—500 workers aged from 21 to 70 years. Kruskal-Wallis test. Spearman’s R correlation analysis.	The maintenance of work–life balance will be indicated by older workers. All employees do not have the same possibilities to take advantage of solutions that provide the support of work-life balance.	The diversity of the answers given by the participants according to the type and state of particpants affiliation was not analyzed.	Work-life balance, workers’assessment, aging workforce
Legislation	[13]	To estimate the impact on the efficiency of the reduction of assortment mechanisms among senior employees.	Italian National Institute of Statistics data from 2009 to 2013. Descriptive statistics. Multivariate regression analysis.	The growth of pension age, as well as limitations on early pension intention, kept older workers at the work without a positive influence on efficiency. More efficient older employees are mroe likely to stay at work in comparison with those who are not as efficient.	The number of employees kept at the work was underestimated. The reform’s influence on the employees’ structure is an additional issue.	aging workforce, pension reforms, labor productivity
	[43]	To investigate the workforce participation and absence among older adults in Sweden.	Data from the Swedish population register. Study sample—workers aged 55–64 years. Descriptive statistics.	The alternation in regulations affected the share of workers associated with illness and disability pension programs. Simultaneously, the share of workers going to early pension has grown.	This study noticed no alternation related to the difference in working-life exit patterns associated with hierarchical and academic positions in the organization.	Workforce participation, older worker, pension, illness benefits
	[20]	To review the expert way of thinking in relation to policies influencing the employment of older adults.	Survey study. Study sample—89 participants aged 50 years or older. Descriptive statistics.	A broad range of policies recommend possibilities for innovation.	There is a sampling bias related to the language and review method. There were no participants from South America, while a few participants from Africa demonstrated about limited Internet access.	Aging workforce, older workers, employment policy, mandatory pension, government answers
	[44]	To investigate whether age and mental capabilities mitigate the connection between job stress and negative affect	Survey study. Study sample—139 workers aged 25–69 years. Descriptive statistics. Correlation and regression analysis. Johnson–Neyman technique.	Cognition mitigated the connection between job stress and negative affect. Crystallized cognition had a large influence on the connection between job stress and negative affect for senior workers. The mitigating influence of fluid cognition was unchanging.	The study did not permit a setup of directionality among variables. Better evaluation of professional features and job requirements is needed.	Job stress, negative affect, older workers

Improve the well-being of older workers. Difficulties that older people experience at work indicates a need for healthcare strategies to adjust the work conditions so that they are suitable for older workforce with decreased physical ability. The authors in [34] identified professions that are dominated by older workers and suggested that work-related hazards (e.g., noise, vibrations, etc.) should be reduced to prevent health problems. Older workers and workers with disabilities can be used as the sources of required skills. Such unutilized workers need to be recruited and well-managed to ensure that their skills are retained [35]. In order to improve the well-being of older workers, the authors in [17] considered the influence of organizational factors, whereas those in [36] examined psychosocial factors at workplace. Unfulfilled prospects for work in old age influenced the prosperity of older workers [41]. Therefore, it is necessary to perform workplace health promotion activities [7].

Promote the lifelong learning. The growth of the aging labour force and emerging technologies change the work environment, generating a need to train older workers to improve their skills. Older workers gain benefits when well-designed training approaches are used. Therefore, the authors in [38] studied the training requirements and work experience, as well as the perception of ideal job features. To encourage technology adoption in the work environment, there is a need to understand how workers study software tools and complete assignments [8]. Therefore, further research should concentrate on developing safer work practices and supporting worker’s productivity and professional longevity [37].

Encourage the late retirement. In order to achieve more successful inclusion of older people into labour market, there is a need for more comprehensive policies and harmonized all-age legislation. This is indicated by the fact that the overall decrease in the share of individuals in pension and disability programs is caused by changes in regulations [43]. In this regard, the authors in [20] studied the factors that affect the aging labour force and the range of current policies that suggest the possible opportunities for innovation. The implications for older workers are related to lifespan earnings, job retention, retirement savings, the possibility of changing jobs, or employment assurance [13,44]. Increasing the pension age should not exacerbate social and health disproportion in the older workers [40]. This is important since many older workers report unequal options to take advantage of solutions for supporting the balance between work and private life [42].

The abovementioned policy implications may be useful from policy making perspective. They could lead to the creation of framework that targets government, the non-governmental sector, private sectors and other stakeholders. However, the creation of such policy framework should take into account many other contributing factors [28] that can be the subject for future research activities. Furthermore, a future research agenda should consider the concept of ageing at work at national level and intensify collaboration at international level. Nevertheless, the following recommendations for governments and other stakeholders can be drawn from this research study:

1. Encourage incentives to extend the working ability in old age;
2. Eliminate age-based discrimination at work along with promotion of gender equality;
3. Invest in education, lifelong learning, health and well-being while increasing the productivity;
4. Improve the working conditions to increase the safety at work and health of workers;
5. Support late retirement along with the increase of life expectancy;
6. Reduce the use of early retirement if workers’ health and work ability are satisfactory.

### 4.2. Study Strengths and Limitations

This study provides a systematic review of articles related to the aging labour force in terms of recent trends and future directions. Additionally, it identifies and evaluates the motivations that drive research on the aging labour force and potential solutions that address the issues related to the aging at work. Sustainable growth and age-based discrimination are recognized as the main motivations to perform the research activities in the given context. On the other hand, policies that stimulate life-long learning are identified as a potential solution for the aging labour force. The additional value of this study lies in its identification of policy implications and recommendations for governments and other stakeholders.

Furthermore, along with this paper, we also provide a Supplementary Materials of all identified relevant articles that can be filtered in terms of different fields to recognize articles for further analysis in a particular subfield. This initial search for a systematic review design may provide useful results on the relevance, practicability, and time needed to carry out a systematic review.

Despite the valuable insights in this study, it suffers from several limitations as well. First, this study took into consideration only three digital libraries, so some relevant articles could be unintentionally omitted from the study because of the specificity of the search strings and the fact that we have not taken into account the non-indexed publishers. However, the size of the searched digital libraries is sufficient, so the obtained results are suitable for the purpose of the study. Additionally, the articles obtained for this study are the results of a search query sent to different search engines with different retrieving and formatting rules from those that are used in the considered libraries. However, we are convinced that the specificities of the publishers’ search engines had no influence on the findings of this study, taking into the account the number of analyzed articles.

Finally, the articles are categorized to provide the quantitative results that show the recent trends and future directions of aging at work, whereas the qualitative results are manually covered to a limited extent to describe the motivation issues and solutions for the aging labour force.

## 5. Conclusions

The aging of the population raises many issues and provides many opportunities. It intensifies the requirement for long-term care, healthcare, and a better-skilled workforce, and increases the demand for age-friendly environments. On the other hand, it enables the contributions of older people to their family, local community, or broader society.

In order to review articles related to the ageing at work in terms of recent trends and future directions, we performed a scoping literature review using an NLP-based framework to automate some of the steps in the PRISMA methodology and quickly identify potentially relevant articles. As a result, starting from over 70 thousand potentially relevant articles, we analyzed in detail about 70 of the most relevant approaches and discussed 24 of them.

We identified that the most prominent works suggest policies and practices that support life-long learning, a workforce that comprises both younger and older workers, and gradual retirement. Approaches like these could be the best response to the globalization issues, reduction of workforce, maintenance of financial independence of the aging workforce, and other social benefits.

Future work could be focused on standardizing approaches to this problem across different countries, supported by different policymakers. The goal should not be to end up with the same approaches in different environments, as this would hardly encompass all cultural, sociological, and economic factors. Instead, we believe that systematically documented and well-thought-out approaches will facilitate the measurement of the results and analysis of causality when investigating benefits and drawbacks.

## Figures and Tables

**Table 1:** The NLP toolkit input parameters: keywords, property groups and properties.

**Keywords**	“active aging at work”, “older adults at work”, “successful aging at work”, “healthy aging at work”, “productive aging at work”
**Property Groups**	Properties
**Motivations**	“deficit”, “discrimination”, “growth”
**Solutions**	“EU policy”, “assistance schemes”, “eligibility criteria", “legislation”, “national policy”

## Supplementary Materials

Supplementary Materials can be found at https://www.mdpi.com/1660-4601/17/20/7659/s1. Table S1: Supplementary list—Data.
